# Before Symptoms Begin: Immune Activation in Preclinical ALS

**DOI:** 10.1002/ana.78174

**Published:** 2026-02-10

**Authors:** Adriano Chiò, Andrea Calvo

**Affiliations:** ^1^ ALS Center, ‘Rita Levi Montalcini’ Department of Neuroscience University of Torino Turin Italy; ^2^ Neurology 1, Azienda Ospedaliero‐Universitaria Città della Salute e della Scienza of Torino Turin Italy

For decades, amyotrophic lateral sclerosis (ALS) was conceptualized primarily as a cell‐autonomous motor neuron disorder, with immune activation viewed as a secondary epiphenomenon. This framework has been fundamentally revised. Accumulating genetic, molecular, and clinical evidence now positions immune dysregulation, both central and peripheral, as a core determinant of disease vulnerability, progression, and outcome.^1^ Within this evolving paradigm, chronic neuroinflammation has emerged as a central and biologically consequential mechanism in ALS and related neurodegenerative diseases.

Converging evidence has consolidated chronic neuroinflammation as a central and biologically consequential mechanism in neurodegenerative diseases, including ALS. In ALS, sustained activation of microglia and astrocytes generates a persistent inflammatory milieu marked by cytokine dysregulation, oxidative stress, and progressive motor neuron injury.^2^ This glia‐driven cascade provides a mechanistic bridge between aging, genetic susceptibility, and selective neuronal vulnerability, reinforcing the view that neuroinflammation is not a passive bystander but an active determinant of disease onset and progression.

Microglia are indispensable for central nervous system (CNS) homeostasis through immune surveillance, synaptic remodeling, and clearance of cellular debris. In ALS, however, prolonged microglial activation becomes maladaptive, fostering chronic neuroinflammation and accelerating neurodegeneration. The long‐standing M1/M2 polarization framework is now widely recognized as an oversimplification. Contemporary transcriptomic and single‐cell analyses instead reveal a continuum of microglial activation states that are dynamic, context‐dependent, and temporally evolving.^3^ In ALS, microglia frequently adopt disease‐associated transcriptional programs characterized by suppression of homeostatic gene networks and upregulation of pathways governing phagocytosis, lipid metabolism, and innate immune signaling, consistent with a stage‐dependent shift from compensatory neuroprotection to overt neurotoxicity.^4^

Beyond the CNS, peripheral immune mechanisms are increasingly acknowledged as integral components of ALS pathobiology. Perturbations across multiple immune compartments, including T lymphocytes, natural killer (NK) cells, monocytes/macrophages, neutrophils, mast cells, complement pathways, and humoral immunity, have been implicated in disease susceptibility and progression.^5^ Nevertheless, a persistent conceptual challenge remains distinguishing primary immune drivers from secondary inflammatory responses to neurodegeneration.

Among adaptive immune pathways, regulatory T cells (Tregs) have emerged as particularly salient modulators of disease trajectory. Tregs preserve immune tolerance by restraining excessive inflammatory signaling. In superoxide dismutase 1 (SOD1) ALS models, higher Treg abundance is consistently associated with delayed disease progression, attenuated glial activation, preserved motor neuron integrity, and prolonged survival.^6^ In patients with ALS, reduced Treg counts and diminished FoxP3 expression correlate with more rapid clinical decline and shorter survival, whereas higher proportions of activated Tregs predict more favorable outcomes.^7^ However, cohort heterogeneity, variability in immune phenotyping methodologies, and the cross‐sectional nature of many studies temper causal inference and underscore the need for standardized, longitudinal immune profiling.

Immune heterogeneity appears to contribute meaningfully to clinical variability in ALS. Accelerated progression and reduced survival have been associated with elevated neutrophil counts and markers of innate immune activation, including the neutrophil‐to‐lymphocyte ratio (NLR) and the systemic immune‐inflammation index (SII).^8^ Immune signatures also vary by sex and age, suggesting patient‐specific immunopathological trajectories. In a prospective study, NK cells and Th2‐differentiated CD4+ central memory T cells have been inversely associated with mortality risk, whereas CD4+ effector memory cells re‐expressing CD45RA (EMRA) and CD8+ T cells have been positively associated with risk of death, emphasizing the dual, context‐dependent roles of peripheral immune populations.^9^ These observations argue against uniform immunomodulatory strategies and instead support precision‐based immune interventions.

C9orf72‐associated ALS and frontotemporal dementia (C9‐ALS/FTD) offer a genetically defined framework in which immune dysregulation is particularly prominent. Microglia in C9‐ALS exhibit disease‐associated transcriptional programs that intensify neuroinflammatory signaling and neuronal susceptibility. Systemically, elevated plasma tumor necrosis factor‐alpha (TNF‐α) and interleukin‐10 (IL‐10), together with lipid alterations such as reduced high density lipoprotein (HDL) in FTD, reflect broader inflammatory perturbations.^10^ However, variability across cytokine and lipid studies underscores the necessity for larger, harmonized cohorts to establish reproducible immune biomarkers.

A major limitation of prior ALS immunology research is its reliance on symptomatic patient cohorts or preclinical animal models, with comparatively limited investigation of presymptomatic individuals. With the exception of studies in asymptomatic carriers of ALS‐associated variants such as C9orf72 and SOD1, the temporal emergence of immune alterations before clinical onset has remained insufficiently characterized.

Within this context, the study by Cao and colleagues^11^ in this issue of *Annals of Neurology* represents a substantive conceptual and methodological advance. Leveraging UK Biobank biospecimens and longitudinal clinical data, the authors examined whether neutrophil‐secreted enzymes predict future ALS risk and whether this relationship is mediated by neurofilament light chain (NfL), a robust biomarker of axonal injury. Three neutrophil‐derived proteins, myeloperoxidase (MPO), S100A12, and matrix metalloproteinase‐9 (MMP‐9), emerged as significant predictors, each with plausible mechanistic links to inflammatory and neurodegenerative pathways.

In Cox proportional hazards models, a 2‐fold increase in MPO was associated with a 1.27‐fold higher annual risk of ALS onset (hazard ratio [HR] 1.27, 95% confidence interval [CI] 1.03–1.56, *p* = 0.027). Comparable risk increments were observed for MMP‐9 (HR 1.22, 95% CI 1.03–1.46, *p* = 0.025) and S100A12 (HR 1.17). These associations remained significant after adjustment for body mass index (BMI), educational attainment, socioeconomic deprivation, smoking, and alcohol consumption. Elevated neutrophil enzyme levels correlated positively with circulating NfL concentrations, and mediation analyses suggested that axonal injury partially accounts for the effects of MPO and S100A12 on ALS risk. Importantly, these associations appeared specific to ALS rather than reflecting nonspecific prodromal changes across neurodegenerative disorders.

Some limitations merit careful consideration. Effect sizes were modest, precluding immediate clinical applicability for individual risk stratification. Furthermore, the mechanistic pathways linking neutrophil activation to central motor neuron degeneration remain inferential, emphasizing the need for experimental validation and replication in independent, well‐characterized cohorts. The observed interaction with BMI, where associations were more pronounced in overweight individuals, raises additional questions regarding metabolic–immune interplay that warrant systematic investigation.

Notwithstanding these constraints, the principal novelty of Cao et al.'s work^11^ lies in demonstrating that peripheral immune perturbations precede clinical ALS in individuals without known pathogenic mutations. By extending immune involvement into the genuinely presymptomatic phase, the study strengthens the case for immune dysregulation as an early and potentially actionable component of ALS pathogenesis. The correlation between neutrophil‐derived enzymes and NfL further enhances translational relevance, positioning these markers as candidate indicators of early axonal injury and disease liability. Although the biomarkers identified in this study are not currently suitable for individual risk prediction in the general population, they highlight neutrophil‐derived enzymes as candidate contributors to early pathogenic processes.

These findings also intersect with broader concepts of immunosenescence, wherein age‐related immune remodeling may modulate vulnerability to neurodegeneration.^12^ Future research integrating immune aging, genetic risk, and longitudinal biomarker trajectories will be essential to refine early detection strategies and therapeutic targeting.

From a translational standpoint, this work reinforces the rationale for targeting peripheral immune pathways in ALS, particularly at preclinical or early symptomatic stages. Active MMP‐9 has recently been shown to be systemically elevated in ALS, identifying it as a plausible therapeutic target.^13^ In parallel, modulation of gut dysbiosis—an emerging driver of peripheral and central inflammation, potentially mediated through NLRP3 inflammasome signaling—represents another promising intervention strategy.^14^ However, successful translation will require biomarker‐guided patient stratification, mechanistic validation, and carefully designed clinical trials to avoid the shortcomings of prior broadly immunosuppressive/immunoregulatory approaches.

In conclusion, immune dysregulation in ALS correlates with greater disease burden, faster progression, and reduced survival, underscoring the systemic pro‐inflammatory nature of the disorder. However, immune alterations alone are unlikely to initiate ALS; rather, they appear to amplify disease progression. Cell‐autonomous motor neuron vulnerability likely serves as the primary trigger, while failure of neuroprotective mechanisms fosters a non–cell‐autonomous, self‐perpetuating inflammatory cascade that accelerates neurodegeneration.^15^ Experimental ALS models have clarified both the initiating neuronal insults and the downstream immune responses that exacerbate motor neuron loss, identifying systemic inflammatory pathways as promising, but not yet validated, therapeutic targets.

Overall, Cao et al.^11^ provide evidence that peripheral immune perturbations can antedate clinical ALS and intersect with early neuroaxonal injury. Their work supports a strategic shift toward presymptomatic and early‐stage therapeutic intervention, where modulation of immune dysregulation may offer the greatest opportunity to alter disease trajectory before irreversible motor neuron loss occurs.

## Author Contributions

Both authors contributed to this commentary. [Correction added on 25 February 2026, after first online publication: Author contribution text has been revised in this version.]

## Potential Conflicts of Interest

Nothing to report.

## References

[1] van Harten ACM , Phatnani H , Przedborski S . Non‐cell‐autonomous pathogenic mechanisms in amyotrophic lateral sclerosis. Trends Neurosci 2021;44:658–668. 10.1016/j.tins.2021.04.008. 34006386 PMC8972039

[2] Calma AD , Pavey N , Menon P , Vucic S . Neuroinflammation in amyotrophic lateral sclerosis: pathogenic insights and therapeutic implications. Curr Opin Neurol 2024;37:585–592. 10.1097/WCO.0000000000001279. 38775138

[3] Ransohoff RM . A polarizing question: do M1 and M2 microglia exist? Nat Neurosci 2016;19:987–991. 10.1038/nn.4338. 27459405

[4] Rodrigues Lima‐Junior J , Sulzer D , Lindestam Arlehamn CS , Sette A . The role of immune‐mediated alterations and disorders in ALS disease. Hum Immunol 2021;82:155–161. 10.1016/j.humimm.2021.01.017. 33583639 PMC7942756

[5] Mimic S , Aru B , Pehlivanoğlu C , et al. Immunology of amyotrophic lateral sclerosis ‐ role of the innate and adaptive immunity. Front Neurosci 2023;17:1277399. 10.3389/fnins.2023.1277399. 38105925 PMC10723830

[6] Garofalo S , Cocozza G , Porzia A , et al. Natural killer cells modulate motor neuron‐immune cell cross talk in models of amyotrophic lateral sclerosis. Nat Commun 2020;11:1773. 10.1038/s41467-020-15644-8. 32286313 PMC7156729

[7] Beers DR , Zhao W , Wang J , et al. ALS patients' regulatory T lymphocytes are dysfunctional, and correlate with disease progression rate and severity. JCI Insight 2017;2:e89530. 10.1172/jci.insight.89530. 28289705 PMC5333967

[8] Grassano M , Manera U , De Marchi F , et al. The role of peripheral immunity in ALS: a population‐based study. Ann Clin Transl Neurol 2023;10:1623–1632. 10.1002/acn3.51853. 37482930 PMC10502618

[9] Cui C , Ingre C , Yin L , et al. Correlation between leukocyte phenotypes and prognosis of amyotrophic lateral sclerosis. elife 2022;11:e74065. 10.7554/eLife.74065. 35287794 PMC8923665

[10] Masrori P , Beckers J , Gossye H , Van Damme P . The role of inflammation in neurodegeneration: novel insights into the role of the immune system in C9orf72 HRE‐mediated ALS/FTD. Mol Neurodegener 2022;17:22. 10.1186/s13024-022-00525-z. 35303907 PMC8932121

[11] Cao W , Yu L , Wang Z , et al. Neutrophil‐secreted enzymes and ALS risk: exploring a potential mechanistic link. Ann Neurol 2026;99:591–605. 10.1002/ana.78161. 41556284

[12] Tsang VSK , Malaspina A , Henson SM . The metabolic intersection between immunosenescence and neuroinflammation in amyotrophic lateral sclerosis. J Inflamm (Lond) 2025;22:36. 10.1186/s12950-025-00460-y PMID: 40866928. 40866928 PMC12392482

[13] Bowser R , An J , Schwartz K , et al. ALS patients exhibit altered levels of Total and active MMP‐9 and several other biomarkers in serum and CSF compared to healthy controls and other neurologic diseases. Int J Mol Sci 2025;26:8900. 10.3390/ijms26188900. 41009467 PMC12469682

[14] Sarkar SK , Gubert C , Hannan AJ . The microbiota‐inflammasome‐brain axis as a pathogenic mediator of neurodegenerative disorders. Neurosci Biobehav Rev 2025;176:106276. 10.1016/j.neubiorev.2025.106276. 40614949

[15] Appel SH , Beers DR , Zhao W . Amyotrophic lateral sclerosis is a systemic disease: peripheral contributions to inflammation‐mediated neurodegeneration. Curr Opin Neurol 2021;34:765–772. 10.1097/WCO.0000000000000983. 34402459

